# Complex Problem Solving in Assessments of Collaborative Problem Solving

**DOI:** 10.3390/jintelligence5020010

**Published:** 2017-03-27

**Authors:** Arthur Graesser, Bor-Chen Kuo, Chen-Huei Liao

**Affiliations:** 1 Psychology and the Institute for Intelligent Systems, University of Memphis, Memphis, TN 38152, USA; 2 Graduate Institute of Educational Information and Measurement, National Taichung University of Education, Taichung 403, Taiwan; kbc@mail.ntcu.edu.tw; 3 Department of Special Education, National Taichung University of Education, Taichung 403, Taiwan; chenhueiliao@gmail.com

**Keywords:** agents, collaborative problem solving, conversation-based assessment

## Abstract

Collaborative problem solving (ColPS) proficiency was developed as a new assessment for the Programme for International Student Assessment (PISA) in the 2015 international evaluation of student skills and knowledge. The assessment framework defined by the PISA ColPS 2015 expert group crossed three major collaboration processes with four problem solving processes that were adopted from the PISA 2012 individual problem solving assessment to form a matrix of 12 specific *skills*. The three major collaboration processes are (1) establishing and maintaining shared understanding; (2) taking appropriate action; and (3) establishing and maintaining team organization. The four problem solving processes are exploring and understanding the problem, representing and formulating the problem, planning and executing strategies, and monitoring and reflecting on the problem-solving activities. This article discusses how the problem-solving dimension was integrated with the collaboration dimension. We also discuss how computer agents were involved in the PISA ColPS 2015 assessment in order to ensure a satisfactory assessment of collaborative problem solving. Examples of the use of agents to assess ColPS are provided in the context of a released PISA item and a project conducted in Taiwan.

## 1. Problem Solving with Agents in Assessments of Collaborative Problem Solving

Collaborative problem solving (ColPS) is one of the important 21st century skills that has attracted interest in international assessments, national assessments of middle and high school students, colleges, business, and the military [1,2,3,4,5]. ColPS is among the other competencies that are valued in the 21st century, such as critical thinking, problem solving, self-regulation, communication and interpersonal skills. ColPS is an essential skill in the home, the workforce, and the community because much of the planning, problem solving, and decision making in the modern world is performed by teams. The success of a team can be threatened by a social loafer, an uncooperative unskilled member, or a counterproductive alliance, whereas it can be facilitated by a strong team member that draws out different perspectives, helps negotiate conflicts, assigns roles, promotes team communication, and guides the team to overcome troublesome obstacles [6,7,8].

It is debatable whether ColPS should be viewed as a new form of intelligence or a critical competency that is valued in the 21st century. The construct clearly is a form of intelligence, but researchers are uncertain whether ColPS has distinctive characteristics that extend beyond individual problem solving. An answer to this question requires conceptual-theoretical deliberation as well as empirical data on whether ColPS has a detectible increment in validity over individual problem solving. In either case, ColPS was selected by the Organisation for Economic Co-operation and Development (OECD) as a new development for the Programme for International Student Assessment (PISA) in the 2015 international survey of student skills and knowledge [4].

With this context in mind, there are two goals of this review article. First, we describe OECD’s framework for PISA ColPS 2015 and some of the context that framed the framework. Second, we provide justification for the use of computer agents in the assessment scenarios. That is, test-takers in the PISA assessment interact with computer agents rather than fellow humans, a step that stirred some discussion in OECD and affiliated assessment communities. This review article describes how agents are involved in the PISA assessment and also an independent study in Taiwan that incorporated the PISA framework. Unfortunately, it is too early to review data on these assessments because the results have not been released in public reports. However, this review paper analyses the construct as it has been conceived by the OECD’s expert group for PISA ColPS 2015.

ColPS differs from individual problem solving (IPS) in ways that may have both positive and negative consequences. ColPS allegedly has advantages over IPS because (a) there is a more effective division of labour; (b) the solutions incorporate information from multiple sources of knowledge, perspectives, and experiences; and (c) the quality of solutions is stimulated by ideas of other group members. There are also potential disadvantages of ColPS to the extent that members (a) waste time with irrelevant discussion; (b) diffuse responsibility in completing tasks; and (c) disagree in a way that paralyzes progress in solving the problem.

The literature is mixed on whether the quality of solutions is better in a group versus a collection of individuals working independently. Problem solving solutions by a group are sometimes better than the sum of the solutions of the individual members [9,10,11]. However, this positive emergence does not always occur for any number of reasons, such as when one person dominates the team or there is wasted effort in non-germane communication. It is also important to recognize that complete cooperation is not always beneficial. For example, better solutions can sometimes emerge when there are productive forms of conflict (e.g., differences in points of view, disagreements). These and other forms of social disequilibrium minimize premature convergence or closure of discussion (e.g., “group think”, [12]) that can sometimes occur to create positive affect [13,14]. Chronic conflict, however, can have serious negative repercussions. The nonlinear impact of conflict (e.g., disagreements) on ColPS success underscores the need to know when to apply the right ColPS skills at the right times.

A core criterion in ColPS assessment is the quality of the solution to a problem. This requires an objective assessment on the results of group activities, which is very different from an assessment of individual learning in collaborative learning environments. Is the solution to a problem in a group better than by a group of independent individuals, after statistically controlling for solutions that would occur from a sampling of individuals and taking the best solution? Answers to this question require a clear-cut specification of whether the individual or the group is the proper unit of analysis in assessments. Focus on the individual may be better for tracking individual performance, providing feedback, and making recommendations. However, focus on the group better assesses the more holistic emergence of the processes in the group as a whole [15]. The individual and the group are both being considered in contemporary assessment models of both ColPS and collaborative learning [16,17]. In the organizational sciences, multi-level theorizing integrates several levels of analysis (i.e., individuals, groups, organizations) and articulates how constructs cut across levels [18]. By moving beyond the traditional uni-level approach, these organizational theories take a more global systems approach that significantly contributes to the theoretical and methodological investigations of complex collaborative activity.

An essential feature of problems in ColPS is *interdependency*. In essence, a single team member cannot solve the problem alone because the solution requires perspectives and actions of multiple team members who handle different parts of the solution. Communication among team members is needed to coordinate the interdependency and to achieve success on many other aspects of collaboration [19,20,21]. That is, communication is essential for organizing the team, establishing a common ground and vision, assigning tasks, tracking progress, building consensus, managing conflict, and a host of other activities in ColPS. It is important for team members to establish shared knowledge [22,23] on the problem goals, mental models, role assignments, progress on subtasks being accomplished, and outcomes at varying levels of grain size. The shared knowledge also needs to accommodate differences in perspectives and the complementarity of team members so the team can succeed as a whole in achieving the group goals. As the problem is solved, the team members need to know who is not delivering on assigned tasks so the group can adjust and find ways to work around the obstacles. This requires monitoring and reflecting on the progress on tasks and on whether particular members are completing their individual tasks. These activities are well beyond those that are needed for individual problem solving. It nevertheless remains an open question as to whether this is a distinctive form of intelligence.

This article describes the assessment of ColPS in PISA 2015 and how the assessment integrates complex problem solving. Developing an assessment of ColPS competency is multifaceted because it requires expertise in such fields as individual problem solving, team science, computer-mediated collaborative work, individual and team cognition, discourse processes, and communication theory. The Collaborative Problem Solving Expert Group (CPEG) adopted this interdisciplinary stance when it developed the framework for PISA ColPS 2015 [4]. It was the first time that a ColPS assessment had been developed for a large-scale international test, so the expert group had to construct a new framework from a diverse and often disconnected set of fields rather than modifying a previous assessment.

In addition to describing the PISA ColPS 2015 framework, this article has a major objective in describing and justifying the use of computer agents in PISA’s assessment of ColPS. We show how ColPS is assessed by having test-takers interact with computer agents through chat interactions, as opposed to interacting with other humans. Although conversational agents have been used to assess and to facilitate collaborative interactions (e.g., [24,25,26,27]), the decision to have the students interact with computer agents during the PISA ColPS 2015 assessment was motivated by important methodological, psychometric, and logistical assessment constraints (see next section). We illustrate how agents are used in the PISA ColPS framework and also data collected in Taiwan, where the PISA framework with agents was used to assess COLPS in their country [28]. It is not possible at this time to present the ColPS items used in the 2015 assessment and to report data from PISA ColPS 2015; the data are currently being analysed and the OECD report has not been released on an assessment that involved approximately 400,000 15-year old students from three to four dozen countries. However, the study in Taiwan provides preliminary findings that show how ColPS can be assessed with agents in a manner that follows the PISA ColPS 2015 framework [4] and that can be validated by individual problem solving.

## 2. Collaborative Problem Solving in PISA 2015

The following definition of ColPS was articulated in the PISA ColPS 2015 [4]: “*Collaborative problem solving competency is the capacity of an individual to effectively engage in a process whereby two or more agents attempt to solve a problem by sharing the understanding and effort required to come to a solution and pooling their knowledge, skills and efforts to reach that solution*”. The unit of analysis for the competency is the individual working with a group rather than the group as a whole. The competency is an assessment on how well the individual interacts with agents during the course of problem solving; this includes achieving a shared understanding of the goals and activities as well as efforts to solve the problem and pooling resources. An agent could be considered either a human or a computer agent that interacts with the student. In both cases, an agent has the capability of generating goals, performing actions, communicating messages, sensing its environment, and adapting to changing environments.

The PISA ColPS 2015 framework [4,25] crossed three major ColPS processes with the four major problem solving processes that were adopted in PISA 2012 for individual complex problem solving [29,30,31]. This resulted in a matrix of 12 specific *skills*. There are three levels of performance (“below”, “at”, or “above” standard) for each of these 12 skills, with associated actions and chat communications that operationally define what it means for the student to be proficient in the skill.

Table 1 presents the skills of the 3 × 4 ColPS Framework [4]. As mentioned, the dimension of problem solving processes contains the same four components as the PISA 2012 framework for individual complex problem solving [31]. The dimension of collaboration incorporates many of the skills identified in models or theoretical analyses of ColPS, such as the team work processing model of the National Center for Research on Evaluation, Standards, and Student Testingteamwork processing model [32], the teamwork model of Fiore and colleagues [21,33,34] and the Assessment and Teaching of 21st Century Skills (ATC21S) [1,2].

### 2.1. The Collaboration Dimension

The three major collaboration processes (called *competencies* in the assessment framework) are (1) establishing and maintaining shared understanding; (2) taking appropriate action; and (3) establishing and maintaining team organization.

(1) **Establishing and maintaining shared understanding.** Team members need to identify what each other knows about the problem (i.e., shared knowledge, common ground [19,22,23]) to identify the perspectives of other agents in the collaboration, and to establish a shared vision of the problem states and activities [13,20,34,35]. They need to establish, monitor, and maintain the shared understanding throughout the problem-solving task by responding to requests for information, sending important information to agents about tasks completed, establishing or negotiating shared meanings, verifying what each other knows, and taking actions to repair deficits in shared knowledge. One important way to accomplish this with digital technologies is to have a *transactive memory*, a system of knowledge about who knows what [36] so that an effective team knows how the knowledge is distributed. In order to acquire a shared mental model, there needs to be a mechanism through which groups collectively record, store, and retrieve information about the status of the problem and individual assignments [37,38,39,40].
(2) **Taking appropriate actions to solve the problem.** Team members must be able to identify the type of ColPS activities that are needed to solve the problem and to follow the appropriate steps to achieve a solution. The actions include taking actions that solve the main substantive problem and follow the framework of complex problem solving [29,30,31]. They also include communication acts, sometimes referred to as team knowledge building [21], such as verifying, ratifying, clarifying, explaining, justifying, negotiating, debating, and arguing with team members.
(3) **Establishing and maintaining group organization.** Team members need to help organize the group to solve the problem by considering the talents and resources of group members as roles are assigned. In line with what Kozlowski and Ilgen [41] categorized as team processes, team members need to follow the rules of engagement for their own roles, monitor the group organization, reflect on the success of the group organization, and help handle communication breakdowns, conflicts, and obstacles. Team members need to take steps to make sure that agents are completing tasks and communicating important information.

### 2.2. Problem Solving Dimension

The problem-solving processes (or competencies) were directly incorporated from the PISA 2012 problem solving framework that targeted individual problem solving [29,30,31]. The four cognitive processes include:

(A) **Exploring and understanding.** This includes interpreting the initial information about the problem and any information that is uncovered during exploration and interactions with the problem.
(B) **Representing and formulating.** Information is selected, organized, and integrated with prior knowledge. Also included are approaches to solving the problem at a global level and identification of relevant strategies and procedures. Graphs, tables, formulae, symbolic representations, and other artefacts may be recruited.
(C) **Planning and executing.** This includes identifying the goal of the problem, setting sub-goals, developing a plan to reach the goal state, and executing the plan. The plans may involve physical actions, social interaction, and communication.
(D) **Monitoring and reflecting**. This involves monitoring steps in the plan to reach the goal state, marking progress, and reflecting on the quality of the progress or solutions.

### 2.3. Matrix of 12 ColPS Skills

The 12 skills in the Table 1 matrix represent a combination of these collaboration and problem solving dimensions. Effective execution of these skills collectively contribute to an overall assessment of ColPS proficiency and can be viewed as a particular form of collaborative intelligence and an important 21st century skill. A satisfactory assessment of ColPS therefore needs to assess the skill levels of test-takers for each of these 12 cells. Some of these skills are reflected in actions that the test-taker performs, such as making a decision by choosing an item on the screen, selecting values of parameters in a simulation, or preparing a requested report. Other skills require acts of communication, such as asking other group members questions, answering questions, making claims, issuing requests, giving feedback on other agents’ actions, and so on. These acts of communication were captured in a chat facility in PISA ColPS 2015.

The PISA ColPS 2015 framework suggested three levels of proficiency for each skill: *Below*, *At*, versus *Above* standard. Individuals *Below* the standard of proficiency are ineffective in advancing group goals because they do not respond to requests for information and to prompts for them to take action, they do not take actions that contribute to achieving group goals, and they perform random or irrelevant actions. Individuals *At* the standard of proficiency are good responsive team members, but do not assertively take the initiative and solve difficult barriers in collaboration. They respond to requests for information and prompts for action, as well as selecting actions that help achieve group goals, but they do not proactively take the initiative in requesting information from the agents, performing unprompted actions, and effectively handling conflicts, changes in the problem situation, and new obstacles to goals. Regarding the problem-solving dimension, they cannot handle very complex problems that have high demands on reasoning and working memory. Individuals *Above* the standard of proficiency meet the *At* threshold, but also proactively take the initiative to solve difficult barriers in collaboration and/or advance relevant solutions to difficult challenges on the problem-solving dimension.

### 2.4. Logistical Considerations

Some logistical considerations influenced how ColPS was assessed in the international assessment. Several dozen countries and languages needed to be assessed, so it was impractical to analyse open ended conversations among team members in a short amount of time with a limited budget. OECD requested a computer-based assessment that measures ColPS skills of individual test-takers in a short time window of two 30-min sessions. The time constraint required that a test-taker would complete four to five different problem solving scenarios within one hour. The test-takers are 15-year-old students who are randomly selected from countries who participate in the PISA assessments. These test-takers would need to interact in a web-based platform at times that could be accommodated by school systems, families, and the community. Computer mediated communication with small groups was technologically available at the time of the assessment, but there were challenges in implementing them in all countries within the sample. It was logistically difficult to organize small groups of test-takers to be networked synchronously at the right times that were acceptable to families, school systems, and the community.

## 3. Computer Agents in the PISA ColPS Assessment

In systems with computer agents, the test-taker interacts with digital team members who generate chat messages and perform actions. The test-taker responds to these messages and actions in addition to other events in the problem scenario. The test-taker interacts with a computer system on the internet rather than other humans so the test-taker can participate in the assessment at a convenient time with available digital facilities.

### 3.1. Justification for Using Computer Agents

The various logistical constraints described in the previous section motivated the use of computer agents instead of teams of humans in the PISA ColPS assessment. However, there were several more important reasons than logistical constraints for using computer agents in the PISA ColPS assessment. Computer agents provide control over the social interaction so that important assessments can be made with consistency and control. Consistency and control are essential requirements of any assessment. These requirements cannot be guaranteed when a small group of humans solve problems together and can meander in many different directions. An adequate assessment needed to include assessment opportunities that cover all 12 cells in the Table 1 matrix. The PISA assessment with agents successfully covered all 12 cells, whereas this could never be guaranteed when a small group of test-takers interact in computer mediated communication.

An adequate psychometric assessment requires a test-taker to interact with different groups and ensembles of team members so that there is a broad distribution of assessment opportunities. Computer agents provided this in multiple scenarios that had judiciously selected sets of team members that generated a broad distribution of chat messages and actions. A strategically generated distribution of assessment opportunities could not be guaranteed in teams of human test-takers. Indeed, the scores of a test-taker would be indeterminate if the test-taker were paired with other humans who do not collaborate and consequently would create a serious form of measurement error.

In summary, computer agents provided a solution to challenges that arose from: (a) the necessity of having multiple teams and problem scenarios per test-taker to obtain reliable assessments in different circumstances (see Table 1); (b) extreme measurement error that would otherwise occur when particular test-takers are assigned to other humans who have unpredictable collaboration difficulties; and (c) logistical difficulties in assembling groups of humans in a timely manner in school systems and communities that have schedule constraints.

The use of agents limited the conversational interactions compared to free chat interactions among humans. Unfortunately, free chat interactions required either an automated analysis of natural language or an expensive process of having experts annotate and analyse the chat interactions. Automated natural language was impractical because the PISA assessment involved dozens of languages, most of which did not have sufficient advances in computational linguistics. Progress has been made in automated analysis of chat interactions in English and a few other languages [16,42,43,44,45], but the precision of these analyses is modest at this point in research and development. The prospects of having experts annotate and analyse the chat interactions would take years [46], which did not fit the time table and budget of PISA assessments.

In addition to the matter of natural language analysis, it would be essential to analyse sequences of conversational turns, actions, and events in conversations that could evolve in any direction in human-human interactions. Patterns of conversation, actions, and events would need to be identified, classified, and mapped onto the cells in the Table 1 matrix. Automated analyses of ColPS and collaborative learning have been developed in previous research projects that analyse the language in computer mediated communication among team members [16,43,45,47]. These research efforts have automatically assessed group cohesion, responsivity of individual team members to a group, and the personality of team members, but they have not yet mapped the chat interactions to the 12 cells in the Table 1 matrix. Moreover, there is no guarantee that all of the cells in the Table 1 matrix would be covered in these analyses of unconstrained chat, which would present issues of incomplete data.

Scepticism has occasionally been expressed on the use of computer agents in the ColPS assessments because there is the concern that the agent approach will not capture the mechanisms of human-human ColPS. However, agents were used for PISA ColPS 2015 after careful consideration of various costs and benefits of human-human versus human-agent interactions, as discussed above. The developers of the PISA ColPS (Educational Testing Service, ETS) conducted pilot testing and a field test on a sample of participants in different countries; ETS and OECD found no significant obstacles to prevent them from completing the PISA ColPS assessment in 2015 on several dozen countries. The results of these analyses were not available to the public when this article was written, but data are expected to be released in 2018. OECD also commissioned a study to compare ColPS assessment with human-human versus human-agent interactions, but these data are not yet available to the public (Greiff, personal communication).

### 3.2. Implementation of Assessments with Agents

When computer agents were created in PISA ColPS, it was important to impose some limitations on the persona of the agents and the types of input in which the test-taker would communicate. Minimalist agents were used without speech, realistic visual depictions, animation, or gestures because those aspects vary among countries and cultures. The agents consisted of chat messages and icons that were culturally neutral. The OECD explicitly requested that personality, emotions, and culture dimensions should not be part of the ColPS assessment because the focus was explicitly on the cognitive aspects of collaborative problem solving.

Team composition would be expected to have an impact on the ColPS of a group, as discussed earlier. For example, test-takers would be handicapped if they were paired with uncooperative agents or agents that ignore them. The test-taker in the PISA ColPS assessment encountered a broad distribution of agents (e.g., helpful, attentive, assertive, negligent, uncommunicative, incorrect) and the system tracked the test-taker’s responses. That being said, there was one constraint that was motivated by a noteworthy cultural consideration. Some cultures do not sanction a low status individual to ask a question, make a request, or even initiate a speech act with a higher status individual. This would place such cultures at a disadvantage that would be reflected in a high differential item functioning index among cultures. Therefore, the roles in the groups had a *symmetrical* structure with respect to status, but could differ with respect to roles [13]. Symmetry of status involves collaboration among peers rather than interactions involving team members with high status differences, boss–subordinate relationships, and teacher-led interactions with students.

As discussed earlier, interdependency is a central property of tasks that are desired for assessing ColPS, as opposed to a collection of independent individual problem solvers. A task has higher interdependency to the extent that entity A cannot solve a problem without actions of entity B. An example consists of jigsaw problems where a group goal requires the accomplishment of a set of tasks (X, Y, and Z), each of which is taken up by a particular team member, and each member has limited access to the other members’ knowledge [9,48]; the puzzle can only be solved when the products of the tasks are pooled and coordinated. Tasks with high interdependency require a coordination among team members that assigns tasks to team members and insures that each member is making adequate progress.

## 4. Scenarios to Illustrate Assessment of ColPS Skills

Two examples are presented in this section in order to illustrate how the 12 skills are assessed through the use of agents in the PISA ColPS 2015 framework. The first example, the *Visit*, is an item that was released by OECD to the public to clarify the PISA ColPS 2015 assessment [49]. The second example was used in a study conducted in Taiwan [28] that adopted the PISA ColPS 2015 assessment framework in order to develop and test their own items.

### 4.1. The Visit: A Released Item for PISA 2015 ColPS

In this unit, a group of international students is visiting a school. The test-taker collaborates with three agent teammates and a faculty advisor (Ms. Cosmo) to plan the visit, assign visitors to guides, and respond to unexpected problems that arise. In Part 1, the test-taker and teammates collaboratively identify an appropriate trip to a point of interest by discussing their preferences, making recommendations, and converging on a final selection. Three alternative sites are considered: a museum of local history, a community open-air market, and an electric car factory. Properties of each site to consider include the travel distance, because it must fit within the time allocation. The Visit is a *constraint satisfaction* problem, a class of problems that is frequently used in individual problem solving assessments [31]. That is, the team needs to converge on a selection that satisfies multiple constraints. Moreover, the team needs to repair a misunderstanding over the hours in which one of the alternative sites is open. Consequently, the problem is classified as a *dynamic* problem rather than a *static* problem because constraints change during the course of solving the problem rather than remaining constant [29,30,31]. Unlike individual problem solving, collaborative skills are needed to solicit and consider criteria for assessing outing options, clarifying statements made by other teammates, correcting misinformation, and prompting teammates to perform their tasks.

The computer screens on the PISA 2015 ColPS assessment support the primary components of collaborative problem solving. Screenshots are available from the field test trial on a released item [49]. The top left of most screen displays have labels that provide an “Introduction” to the problem and “Directions” that the test-taker can access at any point. Below those labels are the list of teammates in the chat, such as George, Rachel, Brad, and You (the test-taker). Below “Who’s in the Chat”, is the chat history, with the possibility of scrolling to earlier points in the chat conversation. Next comes four options on what You (the test-taker) can say next in the conversation. The test-taker selects one of four options, following the standard N-alternative forced-choice item format that is frequently adopted in high scale assessments. To the right of the screen is an area that presents information about the problem state and potential actions that the test-taker can perform. There are icons for the three outing sites (museum of local history, community market, and electric car factory) and a Notepad with problem constraints, to which teammates sometimes contribute. The Notepad plays an important role in externalizing cognition [50] and facilitating collaboration when team members can view the Notepad [51]. In some assessment observations, the test-taker performs actions in this problem information area, such as selecting one of the outing sites. There are a limited number of action options in these observations (in this case three alternatives) so that the N-alternative forced-choice format is followed in the item design. Therefore, there are two types of test-taker input (chat option selection and action option selection) in an N-alternative forced-choice format.

The four chat options in each test-taker’s turn are aligned to one of the cells in the Table 1 matrix. The test-taker is expected to respond to the last chat comment. In one exchange it was Brad, an unproductive off-task contributor: “Who cares? All of these choices are boring. Let’s take our visitors someplace they’ll actually enjoy.” The four chat items from which the test-taker can select are:

(a) You’re right Brad. If none of the choices are any good, let’s go somewhere else.
(b) Brad, you’re right that we want them to enjoy themselves, but we should discuss Ms. Cosmo’s options first.
(c) Ms. Cosmo has no idea what kids like. Rachel, George, do you agree?
(d) Why don’t we take a look at the Town Hall instead?

The credited response is option “b” which acknowledges Brad’s statement but reminds him about the team’s task, provides feedback, and encourages the team to consider the problem constraints. It is the only option that advances the team in solving the problem. This observation addresses cell D1 in the Table 1 matrix: “Monitoring and repairing the shared understanding”. The test-taker not only monitors progress on the team goal but also takes the initiative in getting the team mates on track rather than pursuing a direction that is outside of the problem space.

Problem solving scenarios and assessment observations needed to be carefully composed to allow scores to be computed on each of the 12 cells. One advantage of computer agent assessment is the degree of control over the conversation that could not be possible under human-human interactions. The discourse contributions of three agents (A1, A2, A3) and the digital media (M) can be coordinated so that each [A1, A2, A3, M] sequential display is functionally a single episodic unit (U) to which the human responds through language, action, or silence in a particular human turn (HT). There is a finite-state transition network that alternates between episodic units (U) and human turns (HT), which is formally isomorphic to a dialogue. There can be conditional branching in the state transition network (STN) so that the computer’s generation of U*_n_*_+1_ at turn *n* + 1 is contingent on the state of the human turn HT*_n_* at turn *n*. However, there were a small number of states associated with each human turn (HT*_n_*) in PISA ColPS 2015, with two to five options at each turn, so the finite-state network is not complex.

In the PISA assessment, there is only one score associated with each episodic unit and each episodic unit is aligned with one and only one cell in the Table 1 matrix. These constraints are compatible with the normal psychometric modelling in the world of assessment. Traditional psychometric assessments require a fixed set of items (i.e., episodic units) that all humans experience. Consequently, PISA ColPS 2015 had fixed sequence episodic units (U_1_, U_2_, … U*_m_*) that were distributed throughout the interaction in the problem-solving scenario. The score for each episodic unit was based on the decision of the test-taker for each unit. Moreover, the conversations were finessed so that the conversations would naturally close at the end of each episodic unit by either an agent’s speech acts (e.g., “We should do X, let’s go on”) or an event in the scenario (such as an announcement that a visitor had to immediately return to his home country in the example scenario). After one episodic unit closed, the next unit would systematically appear. Assessment scores were collected for each test-taker for the M episodic assessment units that were distributed among the cells in the Table 1 matrix.

Test-takers who respond randomly to the response options would obviously be considered low on ColPS proficiency as well as the collaboration and problem solving dimensions. An individual may be a good team player and be responsive, but not take the initiative when there are problems (such as a new obstacle that dynamically appears in the problem, an agent who is unresponsive, or an agent that gives incorrect information). A test-taker may take some initiative when there are breakdowns, but not be able to handle very complex cognitive problems. Therefore, the problem complexity is an important factor in the PISA ColPS 2015 assessment and complexity was defined in similar ways as PISA 2012 for individual problem solving. A test-taker who scores high in ColPS proficiency takes the initiative in moving the team to achieve group goals during difficult times (conflicts, incorrect actions, unresponsive team members) and can also handle complex problems with many cognitive components that burden working memory and require reasoning. Episodic units for these situations are needed in order to have adequate ColPS assessment.

### 4.2. An Assessment in Taiwan that Was Based on the PISA ColPS 2015

At this point the results of PISA ColPS 2015 are currently being analysed, so it is too early to report the results of an assessment that included over 40 countries and 400,000 15-year old students throughout the globe. A ColPS proficiency scale with multiple levels has not yet been determined. Meanwhile, however, these researchers [28] conducted their own assessment in Taiwan that adopted the PISA ColPS 2015 assessment framework on the internet with computer agents. Any success they had in creating and testing the materials is consistent with the view that the PISA ColPS framework is sufficiently well specified that it can be replicated by independent researchers. Unfortunately, it is beyond the scope of this review article to summarize all of the results of the Taiwan study, which is in the process of being completed.

The Taiwan ColPS assessment had five tasks entitled Graduation Trip, Water Purification, Slurpee, Game of 25, and Tower of Hanoi. These tasks covered domain knowledge in reading, science, and mathematics. The test-takers were limited to 100 min when completing the five tasks. There were 53,855 grade 9 and 10 students (27,663 boys, 27,192 girls) who participated between October 2014 and February 2015.

The computer interface of the Taiwan assessment was isomorphic to the PISA ColPS 2015 assessment. That is, there was a chat history window, a set of alternative chat options to be selected by the test-taker, and the task information to the right. The input channels of the test-taker included the selection of a chat alternative or performing an action in the task information area of the screen.

The selection of tasks and response alternatives was guided by the Table 1 matrix. The assessment construction process followed the principles of evidence centred design [52]. In order to establish ColPS competency scales, the researchers conducted a multidimensional item response model; more specifically, they applied the multidimensional random coefficients multinomial logit model [53] with partial credit data [54]. The reliability and model fitness of the proposed ColPS assessment were all statistically acceptable [28].

Performance was assessed for both the collaboration dimension and the problem-solving dimension of the ColPS framework. Regarding the collaboration dimension, the competency scores showed the following ordering: Establishing and maintaining shared understanding > establishing and maintaining team organization > taking appropriate action to solve the problem. Regarding the problem-solving dimension, the competency scores showed the following ordering: Exploring and understanding > representing and formulating > planning and executing > monitoring and reflecting. Interestingly, this ordering was consistent with the results of Taiwan in the PISA 2012 on individual problem solving assessment [28]. When expressed in terms of Table 1 codes, 1 > 3 > 2 and A > B > C > D (see Table 1). The competency scores of the 12 individual cells are also reported in [28]. The scores were comparatively low for C2, D2, and D3 and highest for A1, with other cells in between. These results provide important indicators on where training would be helpful in future curricula.

The Taiwan study supports two important claims. First, the PISA ColPS 2015 framework [4] provides a sufficiently well specified assessment that it can be successfully applied to at least one country. Second, the relative ordering of the competency scores for the four problem solving processes were consistent for the PISA ColPS assessment in 2015 and the 2012 PISA assessment of individual problem solving. This result is compatible with the accumulating evidence that problem solving skills are to some extent domain general and transferable to a broad range of applications [55], even though most of the variance is domain specific.

## 5. Summary

This article has reviewed why and how collaborative problem solving (ColPS) proficiency was assessed in the PISA 2015 international evaluation of student skills and knowledge. Three major collaboration processes were crossed with four problem solving processes adopted from the PISA 2012 individual problem solving assessment to form a matrix of 12 specific skills, as shown in Table 1. This article also discusses the rationale of using computer agents in the assessment and some of the concerns of pursuing this approach instead of small groups of human test-takers. The use of agents was illustrated in the context of the released PISA item and a project conducted in Taiwan.

It is widely acknowledged that ColPS is one of the important 21st century skills in discussions of national and international assessments, the curricula of educational institutions, and applied contexts of business, government, and the military [15,21]. The practical value of understanding and assessing ColPS is indisputable.

The question remains, however, whether ColPS is a unique category of intelligence that is fundamentally different from complex problem solving in individuals. ColPS involves distributed cognition with other people in an interdependent activity, with high uncertainty on how the interaction evolves. This presents an inherent dynamic dimension to ColPS, as opposed to the class of static problems where the problem characteristics are declared at the beginning and remain unchanged throughout the course of problem solving. However, the question arises as to whether this dynamic aspect of ColPS is fundamentally different from dynamic problems in individual problem solving [29,30,31]. It is clearly the case that the interdependency requires contributions from other people, but this may or may not be fundamentally different from the dynamic properties of interactive computer simulation environments or realistic problems in the physical world that undergo dynamic changes. Empirical data from PISA ColPS 2015 will shed light on this issue when the results are released by OECD. Will the results of the psychometric analyses show differences between ColPS and independent problem solving, general intelligence, as well as other cognitive proficiencies? Will ColPS have incremental validity in predicting relevant criterion measures over and above other cognitive components? It will take a decade or more of empirical research to answer these questions.

Questions also remain regarding the use of computer agents in the assessment of ColPS. A strong case was made in the PISA ColPS 2015 framework document for the use of agents rather than small teams of test-takers [4]. The agents were selected because they solved a number of measurement and logistical challenges, such as having the test-taker interact with a broad diversity of teams, team members, and problem scenarios in a short, 1-hour, time period. However, questions have been raised as to whether these interactions with computer agents are sufficiently similar to interactions with human test-takers. Comparisons of human-agent and human-human assessments of ColPS are currently being conducted to shed light on this issue. A central question is whether the two forms of interaction can cover the 12 cells in the Table 1 matrix with sufficient reliability and validity. It is an empirical question whether the human-human interactions can cover the landscape of situations that are theoretically needed for an adequate ColPS assessment.

## Figures and Tables

**Table 1:** Matrix of Collaborative Problem Solving Skills for Programme for International Student Assessment (PISA) Collaborative Problem Solving (ColPS) 2015.

Competency	(1) Establishing and Maintaining Shared Understanding	(2) Taking Appropriate Action to Solve the Problem	(3) Establishing and Maintaining Team Organisation
**(A) Exploring and Understanding**	(A1) Discovering perspectives and abilities of team members	(A2) Discovering the type of collaborative interaction to solve the problem, along with goals	(A3) Understanding roles to solve problem
**(B) Representing and Formulating**	(B1) Building a shared representation and negotiating the meaning of the problem (common ground)	(B2) Identifying and describing tasks to be completed	(B3) Describe roles and team organisation (communication protocol/rules of engagement)
**(C) Planning and Executing**	(C1) Communicating with team members about the actions to be/being performed	(C2) Enacting plans	(C3) Following rules of engagement, (e.g., prompting other team members to perform their tasks)
**(D) Monitoring and Reflecting**	(D1) Monitoring and repairing the shared understanding	(D2) Monitoring results of actions and evaluating success in solving the problem	(D3) Monitoring, providing feedback, and adapting the team organisation and roles
